# Supplementary material for: Rational Multi-Modal Transformers for TCR-pMHC Prediction
Source: ArXiv. 2025 Sep 22:arXiv:2509.17305v1. Preprint. [Version 1] (PMC12486057)
Supplement: Supplement 1 [file NIHPP2509.17305v1-supplement-1.pdf]

# Rational Multi-Modal Transformers for TCR-pMHC Prediction (Supplementary)

Jiarui Li
jli78@tulane.edu
Department of Computer Science
Tulane University
New Orleans, Louisiana, USA

Zixiang Yin
zyin@tulane.edu
Department of Computer Science
Tulane University
New Orleans, Louisiana, USA

Zhengming Ding
zding1@tulane.edu
Department of Computer Science
Tulane University
New Orleans, Louisiana, USA

Samuel J. Landry
landry@tulane.edu
Department of Biochemistry and Molecular Biology
Tulane University School of Medicine
New Orleans, Louisiana, USA

Ramgopal R. Mettu
rmettu@tulane.edu
Department of Computer Science
Tulane University
New Orleans, Louisiana, USA

## A Appendix

### A.1 Designed Model Details

We proposed two version models, EGM-1 and EGM-2. Both models leverage the full TCR sequences and epitope as inputs. In the EGM-1, encoders are first applied to TCR A, TCR B, and epitope to extract their features $E_\alpha$, $E_\beta$, and $E_e$ respectively. To enhance the model's ability to capture intra-TCR interactions, we introduce decoders with cross-attention mechanisms between $E_\alpha$ and $E_\beta$, which can be denoted as:

$$D_{\alpha\to\beta} = d(\alpha, E_\beta), \quad D_{\beta\to\alpha} = d(\beta, E_\alpha), \tag{1}$$

where $d$ denotes decoder with cross-attention, and $D_{i\to j}$ denotes use $i$ to do cross-attention with $j$. Then, the co-attention-enhanced TCR features are further processed through cross-attention with the epitope representation, enabling the model to capture interactions between the TCRs and the epitope. This step can be formulated as:

$$D_{e\to\alpha\to\beta} = d(e, D_{\alpha\to\beta}), \quad D_{e\to\beta\to\alpha} = d(e, D_{\beta\to\alpha}). \tag{2}$$

For modeling TCR–epitope interactions, we applied a decoder that incorporates cross-attention between the TCR representations and the epitope, which can be represented following:

$$D_{\alpha\to e} = d(\alpha, E_e), \quad D_{\beta\to e} = d(\beta, E_e). \tag{3}$$

Then, the final probability of TCR–pMHC binding is predicted by concatenating the epitope and TCR representations and passing them through a classification head following:

$$\hat{p}_{\text{bind}} = \sigma([D_{e\to\alpha\to\beta}, D_{e\to\beta\to\alpha}, D_{\alpha\to e}, D_{\beta\to e}]W^T + b), \tag{4}$$

where $[o_1, o_2, ..., o_i]$ denotes concatenate all objects $o_i$, $W^T$ and $b$ denote the weights and bias of linear mapping input to two-dimension outputs for binder and non-binder prediction, and $\sigma$ represents softmax function. EGM-2 is an enhanced version of EGM-1. we introduce interacted intra-TCR information before computing the final TCR–epitope cross-attention, to provide the model with a more comprehensive, global view of the TCR sequences, where the updated $D_{\alpha\to e}$ and $D_{\beta\to e}$ can be represented following:

$$D_{\alpha\to e} = d(\alpha, [E_e, D_{e\to\alpha\to\beta}]), \tag{5}$$

$$D_{\beta\to e} = d(\beta, [E_e, D_{e\to\beta\to\alpha}]). \tag{6}$$

### A.2 ROC-AUCs

**Table 1: The ROC-AUCs of transformer models with various cross-attention designs between epitope and CDR3b.**

| Cross-Attentions | 5-Fold | Test |
|---|---|---|
| Epitope→CDR3b | 0.520±0.008 | 0.520 |
| Epitope→CDR3b + CDR3b | 0.522±0.006 | 0.513 |
| Epitope→CDR3b + Epitope | **0.732±0.006** | 0.504 |
| CDR3b→Epitope | 0.484±0.004 | 0.492 |
| CDR3b→Epitope + CDR3b | **0.718±0.007** | 0.508 |
| CDR3b→Epitope + Epitope | 0.478±0.004 | 0.510 |
| CDR3b↔Epitope | **0.718±0.007** | 0.526 |

The Area Under the Receiver Operating Characteristic Curve (ROC-AUC) scores for cross-attention mechanism exploration and loss strategies comparison. The Table 1 is full table of performance on the test dataset. The Table 2 is the ROC-AUC performance for EGM-1 and EGM-2 with MLM loss or auxiliary losses.

**Table 2: The ROC-AUCs of explanation-guided models with different loss strategies.**

| Loss | 5-Fold | Test |
|---|---|---|
| **EGM-1** | | |
| Classification Only | 0.882±0.006 | 0.755 |
| Classification + MLM | 0.885±0.003 | 0.760 |
| Classification + MLM + MHC | **0.890±0.003** | **0.765** |
| Classification + MLM + TRVJ | 0.885±0.005 | **0.765** |
| **EGM-2** | | |
| Classification Only | 0.880±0.006 | 0.760 |
| Classification + MLM | 0.888±0.003 | 0.765 |
| Classification + MLM + MHC | **0.890±0.002** | **0.771** |
| Classification + MLM + TRVJ | 0.885±0.004 | 0.765 |

## A.3 Training Strategy

The explanation-based model selection strategy is evaluated on EGM-1. The model selected by this strategy shows comparable and stable generalization ability starting from the 350 epoch.

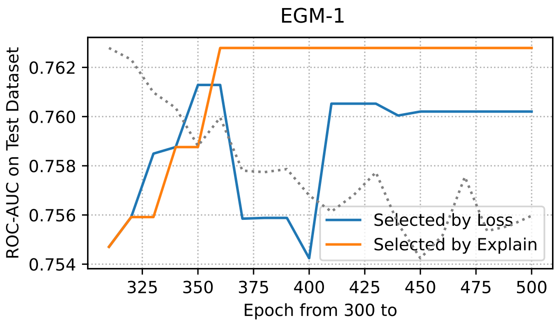


**Figure 1: The ROC-AUC on the independent test dataset for the best models selected either by minimal loss or by highest explanation quality from epoch 300 to 500. From the 350 epoch, explain-based model selection strategy can select the model with better generalization ability in stable.**

## A.4 Statistical Tests for Significance

Because MixTCRpred demonstrates the best performance and generalization ability among the baselines, we use it as the baseline model for significance test. We apply DeLong's test to assess the statistical significance of differences in ROC-AUC between MixTCRpred and our models on both the test dataset and the 5-fold cross-validation results. As shown in the Table 3, all p-values are less than 1E-5, indicating that the ROC-AUC improvements of our models over MixTCRpred are statistically significant.

| Dataset | EGM-0<br>- | EGM-1<br>- | EGM-2<br>- | EGM-2<br>MHC | EGM-2<br>TRVJ |
|---|---|---|---|---|---|
| 5-Fold | $7E-32$ | $8E-65$ | $5E-81$ | $9E-79$ | $2E-94$ |
| Test | $1E-7$ | $1E-8$ | $1E-10$ | $6E-12$ | $1E-10$ |

**Table 3: Statistical tests of significance for ROC–AUC comparison between EGM and TCRMixPred.**

## A.5 Binding Site Hit Rate

The Binding Site Hit Rate (BRHR) table with 0.25 as threshold for the models of input modality selection, analysis of cross-attention mechanism, explain-guided models, and various loss strategies sections. It contains the BRHR analysis based on both positive and negative samples and positive-only samples.

**Table 4: The Binding Region Hit Rate (BRHR) for transformers with various group of CDR input modalities.**

| Modalities<br>Interact with | Epitope<br>TCR A | <br>TCR B | CDR3a<br>Epitope | CDR3b<br>Epitope |
|---|---|---|---|---|
| CDR3b + Epitope | 0.7715 | 0.6682 | - | 0.7386 |
| All CDR3s + Epitope | 0.6929 | 0.6743 | **0.8422** | **0.8222** |
| All CDRs + Epitope | 0.7052 | **0.7369** | **0.7783** | 0.6398 |
| Positive-only Samples Analysis | | | | |
| CDR3b + Epitope | 0.7815 | 0.6636 | - | 0.7286 |
| CDR3s + Epitope | 0.7160 | 0.6635 | **0.8010** | **0.7983** |
| All CDRs + Epitope | 0.7016 | **0.7340** | **0.7953** | 0.6754 |

**Table 5: The Binding Region Hit Rate (BRHR) for explanation-guided models.**

| Modalities | Interact with | EGM-0 | EGM-1 | EGM-2 |
|---|---|---|---|---|
| Epitope | TCR A | 0.7295 | 0.7487 | **0.7986** |
| | CDR1a | 0.7271 | 0.7776 | 0.8138 |
| | CDR2a | 0.7214 | 0.7597 | 0.8091 |
| | CDR3a | 0.7347 | 0.7530 | 0.7864 |
| | TCR B | 0.6040 | 0.7030 | **0.7378** |
| | CDR1b | 0.5844 | 0.6720 | 0.7095 |
| | CDR2b | 0.6402 | 0.7137 | 0.6995 |
| | CDR3b | 0.6159 | 0.7158 | 0.7381 |
| CDR1a | Epitope | 0.7427 | 0.6989 | 0.7372 |
| | TCR B | 0.7080 | 0.6150 | 0.7080 |
| CDR2a | Epitope | 0.7628 | 0.8120 | 0.7372 |
| | TCR B | 0.7299 | 0.8084 | 0.7153 |
| CDR3a | Epitope | 0.8762 | 0.8899 | 0.8659 |
| | TCR B | 0.7600 | 0.7485 | 0.7698 |
| TCR A | Epitope | 0.7969 | 0.7761 | **0.8765** |
| | TCR B | 0.7248 | 0.6786 | 0.6855 |
| CDR1b | Epitope | 0.8212 | 0.8102 | 0.8613 |
| | TCR A | 0.8650 | 0.8595 | 0.9380 |
| CDR2b | Epitope | 0.3996 | 0.4361 | 0.3212 |
| | TCR A | 0.6150 | 0.6113 | 0.6460 |
| CDR3b | Epitope | 0.6152 | 0.5912 | 0.5456 |
| | TCR A | 0.9060 | 0.8893 | 0.9373 |
| TCR B | Epitope | 0.6438 | 0.6840 | 0.6146 |
| | TCR A | 0.6479 | 0.6402 | 0.6170 |
| Positive-only Samples Analysis | | | | |
| Epitope | TCR A | 0.7019 | 0.7456 | **0.7821** |
| | CDR1a | 0.6988 | 0.7661 | 0.8144 |
| | CDR2a | 0.7185 | 0.7580 | 0.8182 |
| | CDR3a | 0.7006 | 0.7409 | 0.7517 |
| | TCR B | 0.6394 | 0.7207 | **0.7341** |
| | CDR1b | 0.6354 | 0.6978 | 0.7043 |
| | CDR2b | 0.6540 | 0.7166 | 0.6708 |
| | CDR3b | 0.6480 | 0.7307 | 0.7328 |
| CDR1a | Epitope | 0.7370 | 0.6743 | 0.6679 |
| | TCR B | 0.7333 | 0.6029 | 0.6642 |
| CDR2a | Epitope | 0.7222 | 0.8057 | 0.7628 |
| | TCR B | 0.6889 | 0.7743 | 0.7299 |
| CDR3a | Epitope | 0.7543 | 0.7610 | 0.7153 |
| | TCR B | 0.6025 | 0.5690 | 0.5833 |
| TCR A | Epitope | 0.7981 | 0.7320 | 0.7404 |
| | TCR B | 0.7309 | 0.7750 | **0.8024** |
| CDR1b | Epitope | 0.7852 | 0.6714 | 0.6569 |
| | TCR A | 0.8111 | 0.5800 | 0.6460 |
| CDR2b | Epitope | 0.3926 | 0.6886 | 0.7409 |
| | TCR A | 0.5963 | 0.6086 | 0.6752 |
| CDR3b | Epitope | 0.4864 | 0.7376 | 0.7007 |
| | TCR A | 0.5315 | 0.6929 | 0.5803 |
| TCR B | Epitope | 0.6457 | 0.6798 | **0.8413** |
| | TCR A | 0.6459 | 0.6809 | **0.7742** |

**Table 6: The Binding Site Hit Rate (BRHR) for transformers with various TCR input modalities.**

| Modalities | Interact with | All CDRs | TCR A + All CDRbs | TCR B + All CDRas | TCRs | TCRs + All CDRs |
|---|---|---|---|---|---|---|
| Epitope | TCR A | 0.7052 | 0.7273 | **0.7592** | 0.7016 | 0.6967 |
| | CDR1a | 0.6885 | 0.7422 | 0.7759 | 0.6907 | 0.7076 |
| | CDR2a | 0.7096 | 0.7667 | 0.8018 | 0.7089 | 0.7234 |
| | CDR3a | 0.7198 | 0.7440 | 0.7820 | 0.7111 | 0.7085 |
| | TCR B | 0.7369 | 0.7462 | **0.7886** | 0.7713 | **0.8102** |
| | CDR1b | 0.7378 | 0.7227 | 0.7603 | 0.7603 | 0.8094 |
| | CDR2b | 0.7383 | 0.7624 | 0.7924 | 0.7729 | 0.7922 |
| | CDR3b | 0.7436 | 0.7582 | 0.8020 | 0.7686 | 0.8047 |
| CDR1a | Epitope | 0.6861 | 0.7099 | 0.6916 | 0.6369 | 0.6460 |
| | TCR B | 0.7044 | 0.7682 | 0.6460 | 0.5292 | 0.6259 |
| CDR2a | Epitope | 0.7664 | 0.7774 | 0.6953 | 0.7810 | 0.7737 |
| | TCR B | 0.7518 | 0.7719 | 0.6715 | 0.7865 | 0.7719 |
| CDR3a | Epitope | 0.7783 | 0.6937 | 0.8330 | 0.6977 | 0.6645 |
| | TCR B | 0.8756 | 0.7567 | 0.8245 | 0.6791 | 0.7086 |
| TCR A | Epitope | - | **0.9069** | - | **0.9033** | 0.8612 |
| | TCR B | - | 0.6466 | - | 0.6319 | **0.6846** |
| CDR1b | Epitope | 0.7354 | 0.7646 | 0.6642 | 0.5821 | 0.7007 |
| | TCR A | 0.7920 | 0.7482 | 0.5712 | 0.4945 | 0.6277 |
| CDR2b | Epitope | 0.7208 | 0.6880 | 0.6661 | 0.6058 | 0.5438 |
| | TCR A | 0.6314 | 0.6533 | 0.7682 | 0.5420 | 0.5839 |
| CDR3b | Epitope | 0.6398 | 0.7555 | 0.6232 | 0.7206 | 0.7728 |
| | TCR A | 0.7832 | 0.9351 | 0.6182 | 0.8125 | 0.8656 |
| TCR B | Epitope | - | - | 0.8869 | 0.9489 | **0.9538** |
| | TCR A | - | - | **0.6990** | 0.6368 | 0.6420 |
| | | | Positive-only Samples Analysis | | | |
| Epitope | TCR A | 0.7016 | 0.7224 | **0.7731** | 0.7412 | 0.7072 |
| | CDR1a | 0.6775 | 0.7385 | 0.7888 | 0.7244 | 0.7166 |
| | CDR2a | 0.7150 | 0.7573 | 0.8123 | 0.7356 | 0.7433 |
| | CDR3a | 0.7176 | 0.7439 | 0.7978 | 0.7476 | 0.7245 |
| | TCR B | 0.7340 | 0.7365 | 0.7965 | 0.7748 | **0.8346** |
| | CDR1b | 0.7571 | 0.7127 | 0.7699 | 0.7516 | 0.8330 |
| | CDR2b | 0.7520 | 0.7455 | 0.8149 | 0.7574 | 0.8169 |
| | CDR3b | 0.7231 | 0.7490 | 0.8134 | 0.7664 | 0.8276 |
| CDR1a | Epitope | 0.7160 | 0.6257 | 0.6957 | 0.5288 | 0.6011 |
| | TCR B | 0.7006 | 0.5936 | 0.6268 | 0.5168 | 0.5197 |
| CDR2a | Epitope | 0.7901 | 0.7281 | 0.7355 | 0.7861 | 0.7163 |
| | TCR B | 0.7747 | 0.7047 | 0.6993 | 0.7861 | 0.6910 |
| CDR3a | Epitope | 0.7953 | 0.6589 | 0.8062 | 0.6663 | 0.6915 |
| | TCR B | 0.8498 | 0.6131 | 0.8472 | 0.5901 | 0.6273 |
| TCR A | Epitope | - | 0.9084 | - | 0.9109 | 0.8545 |
| | TCR B | - | 0.6413 | - | 0.6275 | **0.6806** |
| CDR1b | Epitope | 0.7191 | 0.7836 | 0.6341 | 0.6130 | 0.5899 |
| | TCR A | 0.7994 | 0.7281 | 0.6159 | 0.4880 | 0.4719 |
| CDR2b | Epitope | 0.6883 | 0.6813 | 0.7138 | 0.6082 | 0.5702 |
| | TCR A | 0.6049 | 0.6199 | 0.6812 | 0.5721 | 0.5955 |
| CDR3b | Epitope | 0.6754 | 0.7598 | 0.6525 | 0.6426 | 0.7832 |
| | TCR A | 0.8066 | 0.9555 | 0.5994 | 0.6550 | 0.7360 |
| TCR B | Epitope | - | - | 0.8823 | 0.9476 | **0.9595** |
| | TCR A | - | - | **0.6903** | 0.6371 | 0.6417 |

**Table 7: The Binding Region Hit Rate (BRHR) for transformers with different cross-attention designs between the epitope and CDR3b.**

| Modalities | Interact with | Epitope ↓ CDR3b | Epitope ↓ CDR3b + CDR3b | Epitope ↓ CDR3b + Epitope | CDR3b ↓ Epitope | CDR3b ↓ Epitope + CDR3b | CDR3b ↓ Epitope + Epitope | CDR3b ↕ Epitope |
|---|---|---|---|---|---|---|---|---|
| Epitope | TCR B | 0.7014 | 0.6877 | 0.6427 | **0.7520** | 0.5398 | **0.8382** | 0.7035 |
| | CDR1b | 0.6748 | 0.6635 | 0.6173 | 0.7198 | 0.5009 | 0.8245 | 0.6936 |
| | CDR2b | 0.6999 | 0.6816 | 0.6558 | 0.7541 | 0.5572 | 0.8241 | 0.7099 |
| | CDR3b | 0.7251 | 0.7114 | 0.6628 | 0.7733 | 0.5432 | 0.8504 | 0.7117 |
| CDR3b | Epitope | **0.7960** | **0.7715** | 0.6578 | 0.6280 | 0.6131 | 0.6246 | 0.6064 |
| Positive-only Samples Analysis | | | | | | | | |
| Epitope | TCR B | 0.7080 | 0.7348 | 0.6739 | **0.7638** | 0.4865 | **0.8397** | 0.6839 |
| | CDR1b | 0.6772 | 0.6894 | 0.6420 | 0.7248 | 0.4255 | 0.8359 | 0.6749 |
| | CDR2b | 0.6657 | 0.6414 | 0.6683 | 0.7610 | 0.5021 | 0.8365 | 0.7018 |
| | CDR3b | 0.7211 | 0.7652 | 0.6913 | 0.7886 | 0.4912 | 0.8639 | 0.6940 |
| CDR3b | Epitope | **0.7369** | **0.7093** | 0.6549 | 0.6122 | 0.6013 | 0.6419 | 0.6064 |

**Table 8: The Binding Region Hit Rate (BRHR) for the explanation-guided model with different loss strategies.**

| Loss | MLM | - | ✓ | ✓ | ✓ | - | ✓ | ✓ | ✓ |
|---|---|---|---|---|---|---|---|---|---|
| | Auxiliary | - | - | MHC | V/J | - | - | MHC | V/J |
| Modalities | Interact with | EGM-1 | | | | EGM-2 | | | |
| Epitope | TCR A | 0.6993 | 0.7487 | 0.7810 | **0.8133** | 0.7473 | 0.7986 | **0.8078** | 0.7915 |
| | CDR1a | 0.7166 | 0.7776 | 0.7800 | 0.8418 | 0.7345 | 0.8138 | 0.8164 | 0.8074 |
| | CDR2a | 0.7262 | 0.7597 | 0.7625 | 0.7755 | 0.7783 | 0.8091 | 0.7847 | 0.7746 |
| | CDR3a | 0.7096 | 0.7530 | 0.7718 | 0.8145 | 0.7695 | 0.7864 | 0.7972 | 0.7827 |
| | TCR B | **0.7537** | 0.7030 | 0.6070 | 0.5622 | **0.8061** | 0.7378 | 0.5875 | 0.6778 |
| | CDR1b | 0.7330 | 0.6720 | 0.5942 | 0.5215 | 0.7802 | 0.7095 | 0.5644 | 0.6467 |
| | CDR2b | 0.7445 | 0.7137 | 0.6302 | 0.5807 | 0.8127 | 0.6995 | 0.5930 | 0.6814 |
| | CDR3b | 0.7457 | 0.7158 | 0.6366 | 0.5869 | 0.8036 | 0.7381 | 0.6187 | 0.6851 |
| CDR1a | Epitope | 0.7591 | 0.6989 | 0.7281 | 0.7354 | 0.7062 | 0.7372 | 0.6953 | 0.6971 |
| | TCR B | 0.7500 | 0.6150 | 0.6642 | 0.6606 | 0.6989 | 0.7080 | 0.6150 | 0.5785 |
| CDR2a | Epitope | 0.7737 | 0.8120 | 0.7226 | 0.7500 | 0.7737 | 0.7372 | 0.7354 | 0.7007 |
| | TCR B | 0.7555 | 0.8084 | 0.7099 | 0.7190 | 0.7573 | 0.7153 | 0.7153 | 0.6807 |
| CDR3a | Epitope | 0.8084 | 0.8899 | 0.8446 | 0.8352 | 0.8172 | 0.8659 | 0.8406 | 0.8768 |
| | TCR B | 0.6731 | 0.7485 | 0.7235 | 0.6910 | 0.6664 | 0.7698 | 0.7971 | 0.7454 |
| TCR A | Epitope | **0.8167** | 0.7761 | 0.7958 | 0.7735 | 0.8354 | **0.8765** | 0.8575 | 0.8135 |
| | TCR B | 0.7052 | 0.6786 | 0.6887 | **0.7296** | 0.6770 | 0.6855 | **0.6885** | 0.6686 |
| CDR1b | Epitope | 0.8595 | 0.8102 | 0.8741 | 0.8412 | 0.8558 | 0.8613 | 0.7883 | 0.8358 |
| | TCR A | 0.9343 | 0.8595 | 0.9088 | 0.9051 | 0.9015 | 0.9380 | 0.7847 | 0.9124 |
| CDR2b | Epitope | 0.3066 | 0.4361 | 0.3248 | 0.3522 | 0.3686 | 0.3212 | 0.4836 | 0.3467 |
| | TCR A | 0.6478 | 0.6113 | 0.6186 | 0.6332 | 0.6332 | 0.6460 | 0.5931 | 0.6369 |
| CDR3b | Epitope | 0.5370 | 0.5912 | 0.5626 | 0.5717 | 0.5665 | 0.5456 | 0.6922 | 0.5754 |
| | TCR A | 0.9349 | 0.8893 | 0.9322 | 0.9224 | 0.9164 | 0.9373 | 0.8549 | 0.9361 |
| TCR B | Epitope | 0.6039 | **0.6840** | 0.6215 | 0.6325 | 0.6293 | 0.6146 | **0.7562** | 0.6240 |
| | TCR A | 0.6125 | **0.6402** | 0.6252 | 0.6275 | 0.6300 | 0.6170 | **0.6389** | 0.6204 |
| Positive-only Samples Analysis | | | | | | | | | |
| Epitope | TCR A | 0.6876 | 0.7456 | 0.7967 | **0.8132** | 0.7533 | 0.7821 | **0.8166** | 0.7581 |
| | CDR1a | 0.6978 | 0.7661 | 0.8071 | 0.8147 | 0.7428 | 0.8144 | 0.8240 | 0.7643 |
| | CDR2a | 0.7119 | 0.7580 | 0.7740 | 0.7787 | 0.7940 | 0.8182 | 0.7928 | 0.7660 |
| | CDR3a | 0.6931 | 0.7409 | 0.7841 | 0.7892 | 0.7734 | 0.7517 | 0.7972 | 0.7233 |
| | TCR B | **0.7427** | 0.7207 | 0.5951 | 0.5748 | **0.8075** | 0.7341 | 0.5817 | 0.7018 |
| | CDR1b | 0.7353 | 0.6978 | 0.5711 | 0.5508 | 0.7977 | 0.7043 | 0.5730 | 0.6724 |
| | CDR2b | 0.7383 | 0.7166 | 0.6095 | 0.5868 | 0.8142 | 0.6708 | 0.5929 | 0.6761 |
| | CDR3b | 0.7328 | 0.7307 | 0.6132 | 0.6033 | 0.7995 | 0.7328 | 0.6145 | 0.7011 |
| CDR1a | Epitope | 0.7194 | 0.6743 | 0.6429 | 0.7162 | 0.6209 | 0.6679 | 0.6905 | 0.6500 |
| | TCR B | 0.7194 | 0.6029 | 0.6116 | 0.6532 | 0.5714 | 0.6642 | 0.6488 | 0.5458 |
| CDR2a | Epitope | 0.7222 | 0.8057 | 0.8036 | 0.6982 | 0.8407 | 0.7628 | 0.7619 | 0.7250 |
| | TCR B | 0.7250 | 0.7743 | 0.7589 | 0.6892 | 0.8077 | 0.7299 | 0.7470 | 0.6917 |
| CDR3a | Epitope | 0.6907 | 0.7610 | 0.7567 | 0.7132 | 0.7358 | 0.7153 | 0.7044 | 0.7319 |
| | TCR B | 0.6102 | 0.5690 | 0.6890 | 0.6697 | 0.6699 | 0.5833 | 0.5823 | 0.6236 |
| TCR A | Epitope | **0.8380** | 0.7320 | 0.6071 | 0.6014 | **0.7498** | 0.7404 | 0.6307 | 0.5508 |
| | TCR B | 0.6879 | 0.7750 | 0.7828 | **0.8250** | 0.6841 | 0.8024 | 0.8086 | **0.8321** |
| CDR1b | Epitope | 0.6528 | 0.6714 | 0.6920 | 0.6396 | 0.6896 | 0.6569 | 0.7113 | 0.6667 |
| | TCR A | 0.5361 | 0.5800 | 0.6562 | 0.6171 | 0.5604 | 0.6460 | 0.6994 | 0.6125 |
| CDR2b | Epitope | 0.7917 | 0.6886 | 0.7723 | 0.7207 | 0.7335 | 0.7409 | 0.6756 | 0.6625 |
| | TCR A | 0.6583 | 0.6086 | 0.6964 | 0.5090 | 0.6648 | 0.6752 | 0.5357 | 0.5833 |
| CDR3b | Epitope | 0.8639 | 0.7376 | 0.6823 | 0.6156 | 0.5537 | 0.7007 | 0.7173 | 0.6986 |
| | TCR A | 0.6986 | 0.6929 | 0.6868 | 0.5083 | 0.5537 | 0.5803 | 0.6409 | 0.6361 |
| TCR B | Epitope | **0.8144** | 0.6798 | 0.7840 | 0.7308 | 0.7606 | **0.8413** | 0.7910 | 0.6896 |
| | TCR A | 0.6709 | 0.6809 | 0.7339 | **0.7377** | 0.6627 | **0.7742** | 0.7140 | 0.7515 |

Received 07 July 2025; revised 10 September 2025; accepted 27 August 2025
